# Photocatalytic CO_2_ reduction to hydrocarbons using FPS@Ph-g-C_3_N_4_-Ho_3_Fe_5_O_12_ under visible-light irradiation

**DOI:** 10.1039/d5ra09226d

**Published:** 2026-07-02

**Authors:** Guang Hao, Na Meng, Yike Wang, Li Gao, Rahele Zhiani

**Affiliations:** a School of Life and Health Management, Shenyang City University Shenyang 110112 China hedgehog2000@163.com; b School of Intelligence and Engineering, Shenyang City University Shenyang 110112 China; c New Materials Technology and Processing Research Centre, Neyshabur Branch, Islamic Azad University Neyshabur Iran R_Zhiani2006@yahoo.com

## Abstract

The selective conversion of CO_2_ into hydrocarbon fuels through photocatalytic methods offers a sustainable route for carbon utilization and energy storage. In this study, a novel hybrid photoelectrocatalyst, FPS@Ph-g-C_3_N_4_-Ho_3_Fe_5_O_12_, was developed to enhance CO_2_ reduction efficiency under visible light assisted electrochemical conditions. The integration of g-C_3_N_4_ with the Ho_3_Fe_5_O_12_ garnet and FPS support enables synergistic improvements in light harvesting, charge separation, and surface reactivity. The g-C_3_N_4_ layer provides a high surface area conductive interface, promoting active site availability and facilitating multi electron transfer processes crucial for hydrocarbon formation. This hierarchical structure effectively drives the reduction of CO_2_ into methane and higher order hydrocarbons through mechanisms involving deoxygenation and C–C coupling. The evolution of product profiles over time provides insights into the reaction pathways and intermediate species. The results highlight the potential of FPS@g-C_3_N_4_-Ho_3_Fe_5_O_12_ as a robust and efficient system for visible light driven CO_2_ conversion toward valuable hydrocarbon fuels.

## Abstract 2

A hierarchical FPS-supported Ph-g-C_3_N_4_/Ho_3_Fe_5_O_12_ heterostructure enables efficient visible light driven CO_2_ conversion into CH_4_ and C_2_⁺ hydrocarbons *via* enhanced charge separation and C–C coupling pathways.

## Graphical abstract

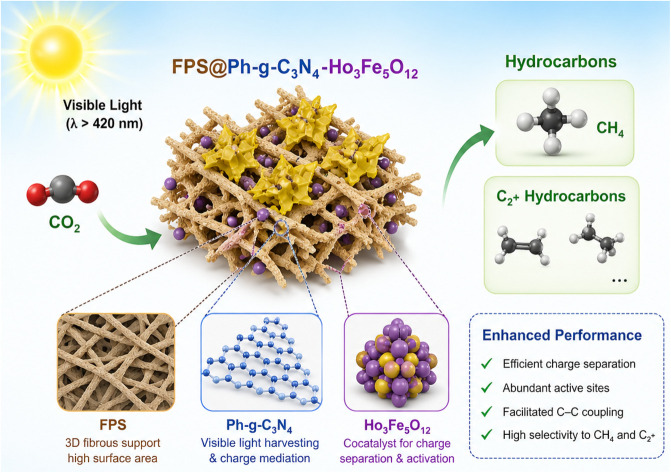

## Introduction

A wide range of materials have been explored for CO_2_ photoreduction and electroreduction using strategies based on structural design, electronic compatibility, and light absorption.^1^ High throughput computational screening has enabled the identification of thousands of potential photocathodes with favorable properties such as stability, visible-light responsiveness, and fuel compatible band structures.^2–4^ Various engineered semiconductors including nanostructured metal organic frameworks, core shell composites, and bimetallic systems have shown selectivity toward products like methane, methanol, ethylene, ethanol, and acetaldehyde under visible light or electrochemical conditions.^5^ Enhanced performance has been observed through approaches such as nanoparticle decoration, heterojunction design, and surface defect engineering, even in the absence of sacrificial agents.^6,7^

The conversion of CO_2_ into multi carbon (C_2_^+^) value added products remains a major challenge because it involves complex multi electron transfer steps and sluggish C–C bond forming kinetics.^8^ Hybrid photocatalysts, including graphene modified TiO_2_, have exhibited enhanced performance in reducing CO_2_ to C_2_H_6_ and CH_4_ in the presence of water vapor. Further improvements in selectivity toward C_2_^+^ hydrocarbons have been achieved by introducing trace amounts of metals such as cobalt and copper.^9–11^ Advanced nickel based catalysts have also shown strong stability during dry reforming by mitigating carbon deposition.^12^ Despite these developments, efficient and selective C–C coupling to produce molecules like ethylene and acetylene remains limited.^13^ Recent approaches concentrate on converting C_1_ intermediates into higher energy C_2_^+^ compounds such as alcohols and olefins through optimized photocatalytic systems.^14,15^

Photocatalytic CO_2_ reduction typically follows three primary pathways formaldehyde, carbene, and the less frequently observed glyoxal route.^16^ The specific reaction mechanism is dictated by whether hydrogenation or deoxygenation processes prevail.^17^ In the hydrogenation pathway, CO_2_ undergoes stepwise reduction to yield formic acid, formaldehyde, methanol, and eventually methane.^18^ Conversely, the deoxygenation route generates intermediates such as CO, carbon centered radicals, and methyl species prior to methane formation. Recent research highlights the critical role of elucidating C–C coupling pathways to improve the selectivity toward C_2_^+^ products.^19^ Moreover, bicarbonate has emerged as an attractive alternative carbon source to gaseous CO_2_, offering advantages for capture and conversion under mild conditions. Protonation mechanisms involving electron transfer and hybrid intermediate species further influence overall selectivity and catalytic efficiency.^20^ In summary, precise identification and regulation of key intermediates are vital for designing next generation photocatalytic systems capable of sustainably producing liquid fuels and other value added multi carbon products.^21–24^ Its moderate surface area, electronic conductivity, and unique band structure make it suitable for use in supercapacitors, batteries, hydrogen storage, and fuel cells. In such devices, g-C_3_N_4_ can function as an electrode material or catalyst support, enhancing capacity, stability, and overall performance.^25^ Similarly, graphene oxide, due to its oxygen containing functional groups, offers strong cation binding ability and colloidal stability, making it a valuable component in composite materials for advanced energy storage systems. Its chemical versatility also enables the formation of reduced graphene oxide composites with enhanced electrochemical properties.^26^

We have engineered a fibrous nanostructure composed of phosphorus and silica. Mesoporous materials such as FPS are widely chosen in nanocatalysis due to their versatile functional properties.^27^ The high surface area of these materials arises from its dendrimeric silicon and phosphorus fibers, combined with distinctive channel structures, making FPS a unique material.^28^ The addition of a surfactant to FPS resulted in the development of a new mesoporous silica structure composed of dendrimeric phosphate, silicon, and dioxide strands.^29^ This material may serve as an effective support in adsorption and catalytic applications. FPS provides a high surface area, enhancing reactant access to the active sites.^30^

Magnetic nanoparticles have recently garnered significant interest from researchers due to their advantageous properties. When an external magnetic field is applied, these properties enable the efficient separation of catalytic compounds from chemical mixtures, facilitating their recovery. The ease of separation *via* magnetic stimulation, combined with their large surface to volume ratio, gives MNPs the potential to purify contaminated water. MNPs can demonstrate adsorption properties during the examined process and act as magnetically directed carriers for absorbent materials attached to their surface layer. Rare-earth orthoferrites with the formula RFeO_3_ (R = La–Lu) have attracted considerable attention as magnetic materials due to their dependable performance and exceptional stability.^31^ Among these orthoferrites, holmium orthoferrite has received particular attention. It possesses characteristics such as functioning as a visible light photoactivator,^32^ a potential multifunctional magnetic material operable at ambient temperatures,^33^ and an antiferromagnet that exhibits a significant spin realignment effect.^34^ Nonetheless, the application of holmium orthoferrite in antibiotic adsorption processes has not yet been investigated, despite its promising magnetic properties. In contrast, magnetic oxide composites offer the potential for desirable magnetic properties, an extensive surface area, abundant active sites, and a strong capacity for surface adsorption.^35^ Previously, a range of magnetic nanocomposites has been employed to remove or adsorb both non-biological and biological contaminants from aqueous solutions.^36,37^ Ferrite garnets represent another class of magnetic materials characterized by inequivalent spin sublattices that are connected through antiferromagnetic interactions.^38^ Ferromagnetic rare earth iron garnets within this category have attracted significant attention due to their distinctive properties, such as magneto caloric effects,^39^ as well as magneto dielectric and magneto optical behaviors.^40^

Considering the promising properties of ferrite based materials, particularly their magnetic recoverability, structural stability, and catalytic potential, the integration of rare earth ferrites with conductive 2D materials has emerged as an effective approach to enhance photocatalytic performance. In this context, the design of a hybrid photoelectrocatalyst combining Ho_3_Fe_5_O_12_ with graphitic carbon nitride (g-C_3_N_4_) on a FPS offers synergistic advantages, including improved CO_2_ adsorption, efficient light harvesting, and enhanced charge separation. These features are expected to significantly boost the photocatalytic conversion of CO_2_ into valuable hydrocarbon products. Therefore, this study aims to investigate the performance and underlying mechanism of CO_2_ reduction using FPS@g-C_3_N_4_-Ho_3_Fe_5_O_12_ as a multifunctional hybrid catalyst under visible light photocatalytic conditions.

## Experimental section

### Synthesis of FPS

To prepare the fibrous phosphosilicate support, 3.42 g of tetraethyl orthosilicate (TEOS) and 4.01 g of tripolyphosphate (TPP) were dissolved in a mixture of 26 mL cyclohexane and 1.7 mL 1-pentanol under continuous stirring. In a separate beaker, 1.8 g of cetylpyridinium bromide (CPB) and 1.1 g of urea were completely dissolved in 27 mL of deionized water. This aqueous solution was then added dropwise to the TEOS/TPP organic mixture. The resulting biphasic system was stirred vigorously at room temperature for 0.9 hours to initiate the formation of the mesostructured hybrid material. The reaction mixture was later subjected to subsequent processing steps, including aging, separation, and drying (details may follow in the next paragraph if available).

### Synthesis of Ph-g-C_3_N_4_

A mixture composed of dicyandiamide (2.0 g) and 4-cyanopyridine (150 mg) was synthesized by dissolving the components in a solvent system containing ethanol (10 mL) and deionized water (10 mL). The solution was stirred vigorously at 65 °C for a duration of 90 minutes. Following this step, the resulting mixture was subjected to rotary evaporation under reduced pressure to remove the solvents efficiently. After solvent removal, the obtained solid was thermally treated at 500 °C for 4 h under air atmosphere to induce polymerization and form Ph-g-C_3_N_4_.

### Synthesis of FPS@Ph-g-C_3_N_4_

The synthesis of FPS@Ph-g-C_3_N_4_ was initiated by dispersing FPS (2.0 mmol) and phenyl modified graphitic carbon nitride (Ph-g-C_3_N_4_, 60 mmol) in 15 mL of anhydrous ethanol under vigorous magnetic stirring. The mixture was then refluxed for 16 hours to facilitate chemical interaction and anchoring of Ph-g-C_3_N_4_ onto the FPS surface. Following completion of the reaction, the ethanol solvent was removed under reduced pressure using a rotary evaporator. The remaining concentrated residue was then dispersed in deionized water to form a homogeneous suspension. Subsequently, precipitation was induced by the addition of a mixed ethanol–tetrahydrofuran solvent system. The resulting solid was collected *via* filtration and finally dried under vacuum at 100 °C to yield the FPS@Ph-g-C_3_N_4_ nanocomposite.

### Synthesis of FPS@Ph-g-C_3_N_4_-Ho_3_Fe_5_O_12_ nanocomposite

In a typical procedure, 0.9 mmol of freshly prepared holmium nitrate pentahydrate (Ho(NO_3_)_3_·5H_2_O), 4.7 mmol of iron(iii) nitrate nonahydrate (Fe(NO_3_)_3_·9H_2_O), and 41 mg of the previously synthesized FPS@Ph-g-C_3_N_4_ were dispersed in an aqueous solution of polyethylene glycol (PEG) under constant stirring. The mixture was stirred at room temperature for 30 minutes to ensure homogeneous distribution of the metal precursors over the FPS matrix. Subsequently, the reaction mixture was heated to 117 °C and maintained at this temperature for 1.7 hours to promote the formation of the Ho_3_Fe_5_O_12_ phase. After cooling to room temperature, the resulting solid product was magnetically separated, thoroughly washed with acetone and distilled water to remove unreacted species, and dried overnight at 50 °C in an oven. Finally, the dried material was calcined in ambient air at 510 °C for 1.5 hours to yield the crystalline FPS@Ph-g-C_3_N_4_-Ho_3_Fe_5_O_12_ nanocatalyst.

### CO_2_ photocatalytic reduction procedure

Before initiating the reaction, the airtight photoreactor was tested for leakage at a pressure of 150 000 Pa over several hours and subsequently purged with high purity helium to remove residual gases. High purity CO_2_ gas (99.9%) was introduced at a controlled flow rate of 15 mL min^−1^ using a mass flow controller. The CO_2_ stream was passed through an aqueous 0.5 M NaOH solution to achieve partial saturation with moisture. The reaction chamber contained the synthesized FPS@Ph-g-C_3_N_4_-Ho_3_Fe_5_O_12_ photocatalyst, uniformly dispersed in the photoreactor. Following irradiation with visible light, gas phase products were withdrawn using a gas tight syringe and injected into a gas chromatography (GC) system for qualitative and quantitative analysis of the CO_2_ reduction products.

## Results and discussion

**Scheme 1:**
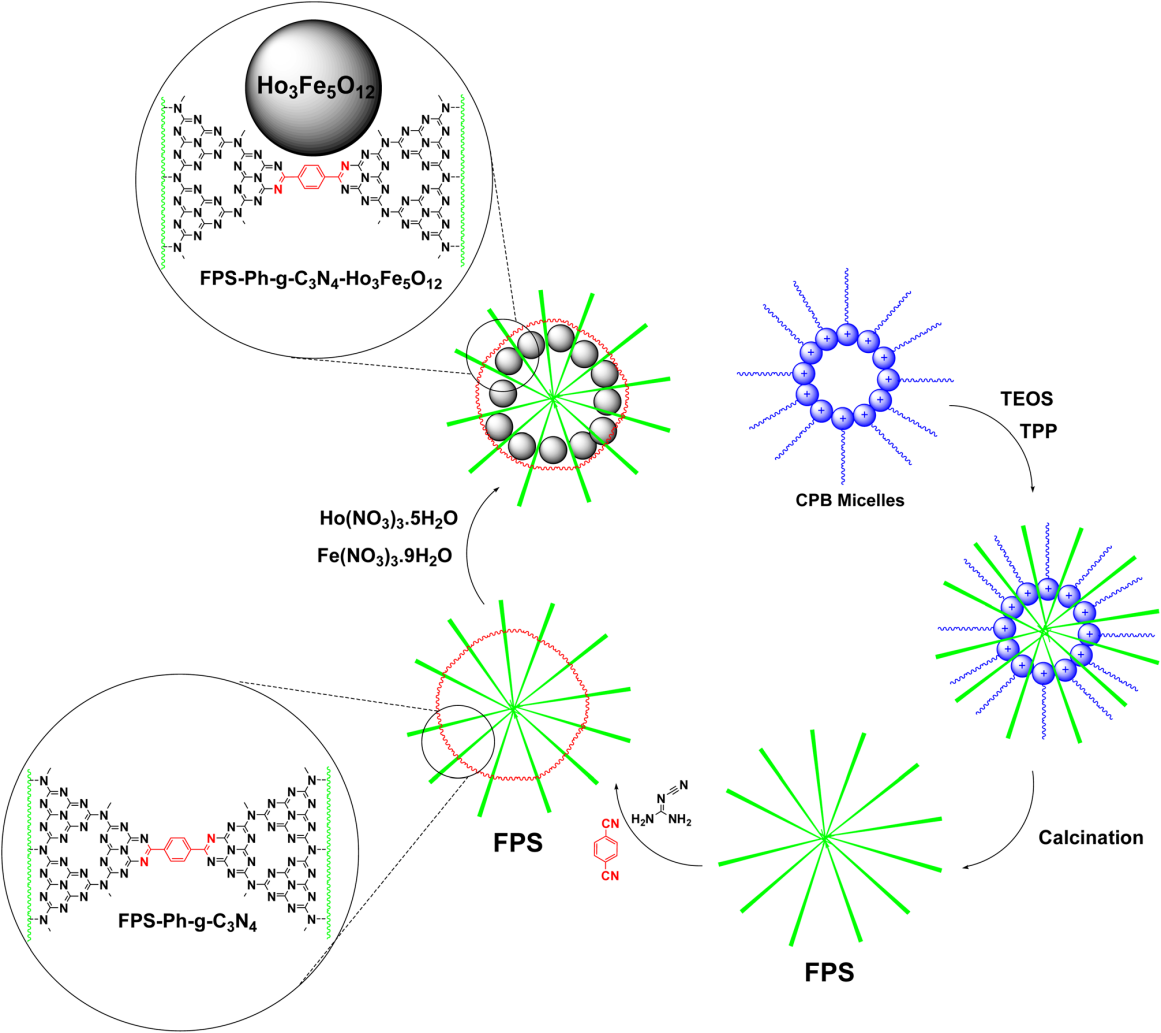
Blueprint of the formulation process for FPS@Ph-g-C_3_N_4_-Ho_3_Fe_5_O_12_.

The synthesis process of the FPS@Ph-g-C_3_N_4_-Ho_3_Fe_5_O_12_ nanocomposite is illustrated schematically in Scheme 1. This composite was fabricated through a practical multi-step strategy involving the integration of a FPS, phenyl modified graphitic carbon nitride (Ph-g-C_3_N_4_), and Ho_3_Fe_5_O_12_ nanoparticles. Initially, the FPS structure provided a high surface area framework enriched with hydroxyl and oxygen containing functional groups, enabling strong interaction with Ph-g-C_3_N_4_ through hydrogen bonding and π–π stacking. The deposition of Ph-g-C_3_N_4_ onto the FPS substrate enhanced the surface conductivity and provided anchoring sites for subsequent Ho_3_Fe_5_O_12_ growth. Upon incorporation of Ho_3_Fe_5_O_12_, uniform dispersion of the ferrite phase was achieved on the FPS@Ph-g-C_3_N_4_ matrix, facilitated by the nucleation supporting role of the modified carbon nitride layer. The resulting composite exhibited a well integrated architecture, combining visible light harvesting, catalytic active sites, and efficient charge transfer pathways essential for photocatalytic CO_2_ reduction.

**Fig. 1:**
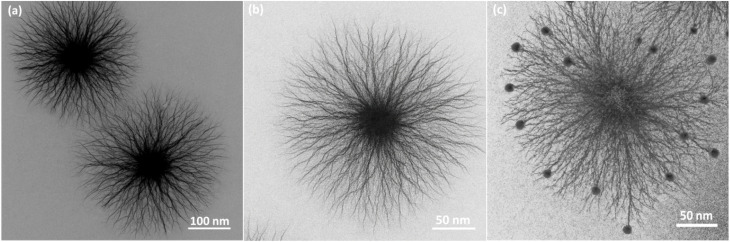
TEM illustrations of (a) FPS, (b) FPS@Ph-g-C_3_N_4_, and (c) FPS@Ph-g-C_3_N_4_-Ho_3_Fe_5_O_12_.

To elucidate the morphological and structural characteristics of the prepared catalyst, transmission electron microscopy (TEM) analysis was conducted (Fig. 1). The images reveal the formation of a well defined three dimensional dendritic fibrous framework in the FPS@Ph-g-C_3_N_4_-Ho_3_Fe_5_O_12_ composite, featuring an interconnected porous network that can facilitate efficient mass transfer and improved accessibility of reactant molecules. Moreover, the TEM observations indicate that Ho_3_Fe_5_O_12_ nanoparticles are homogeneously dispersed throughout the fibrous structure, with a predominantly spherical morphology and particle sizes in the range of approximately 18–23 nm. The absence of noticeable aggregation suggests strong interfacial interactions between the ferrite phase and the FPS support. In some regions, the appearance of larger domains may be associated with slight particle agglomeration or the integration of nanoparticles within the fibrous matrix. Due to the intrinsically low electron contrast and ultrathin layered nature of g-C_3_N_4_, its direct visualization in conventional TEM images is difficult.

**Fig. 2:**
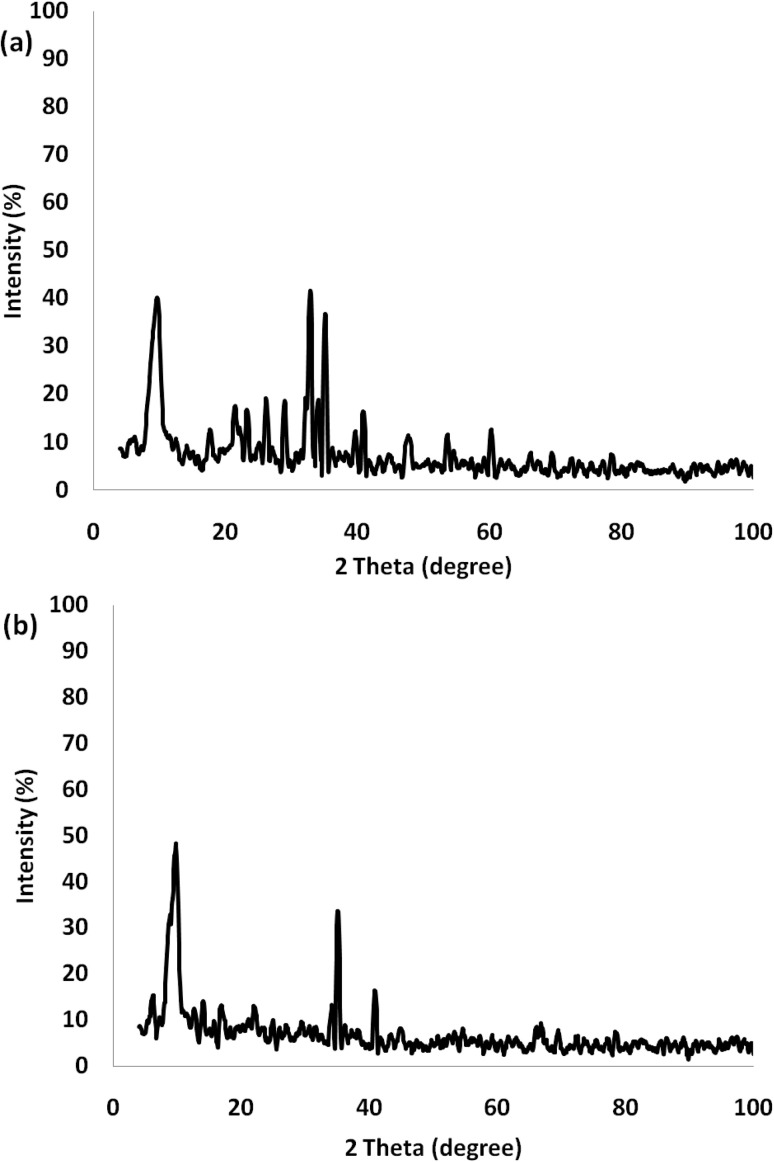
XRD arrangement of (a) FPS, and (b) FPS@Ph-g-C_3_N_4_-Ho_3_Fe_5_O_12_ MNPs.

**Fig. 3:**
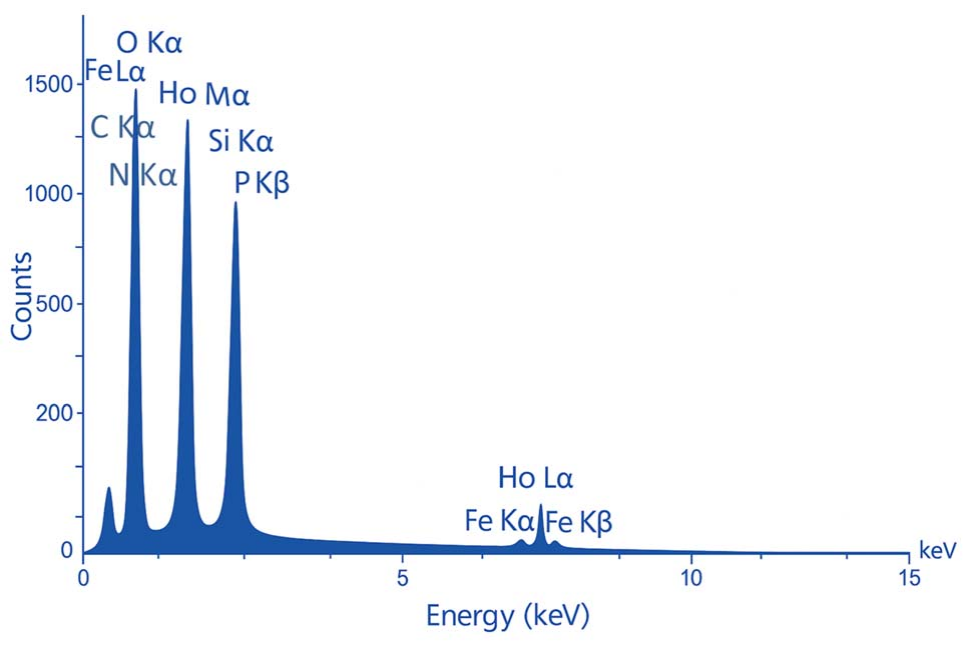
EDX analysis of FPS@Ph-g-C_3_N_4_-Ho_3_Fe_5_O_12_ MNPs.

Fig. 2 illustrates the XRD patterns of both FPS and FPS@Ph-g-C_3_N_4_-Ho_3_Fe_5_O_12_ magnetic nanoparticles (MNPs). The diffraction peaks observed for FPS align well with previously reported crystalline signatures, validating the structural consistency of the synthesized support material. In contrast, the FPS@Ph-g-C_3_N_4_-Ho_3_Fe_5_O_12_ sample exhibits additional reflections that correspond to the Ho_3_Fe_5_O_12_ phase, as confirmed by matching with JCPDS card no. 01-074-1479, thereby verifying the successful deposition of Ho_3_Fe_5_O_12_ crystals onto the FPS framework. A broad peak in the 2*θ* range of 20°–30° further indicates the presence of amorphous silica. To complement the structural analysis, energy dispersive X-ray spectroscopy (EDX) was performed, revealing the presence of Fe, O, N, C, Ho, Si, and P (Fig. 3), thereby confirming the intended elemental composition of the nanocomposite.

**Fig. 4:**
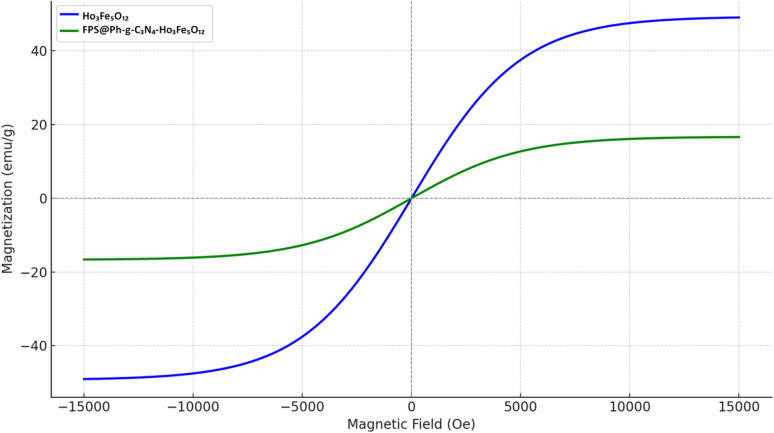
Magnetic hysteresis loops of Ho_3_Fe_5_O_12_ and FPS@Ph-g-C_3_N_4_-Ho_3_Fe_5_O_12_ nanocomposites at 300 K.

The magnetic properties of the synthesized MNPs were investigated using a Vibrating Sample Magnetometer (VSM). The hysteresis curves obtained at 300 K revealed negligible remanent magnetization, confirming the paramagnetic behavior of the nanocomposite (Fig. 4). According to the VSM measurements, the saturation magnetization values for pure Ho_3_Fe_5_O_12_ and the FPS-Ho_3_Fe_5_O_12_ hybrid nanostructure were approximately 49.3 and 16.7 emu g^−1^, respectively. The paramagnetic nature of the nanocomposite, coupled with its substantial magnetization, allows for rapid magnetic response under an external field, with immediate relaxation upon field removal. These magnetic characteristics underscore the nanocomposite's suitability for magnetically assisted targeting, separation, and recovery applications.

**Fig. 5:**
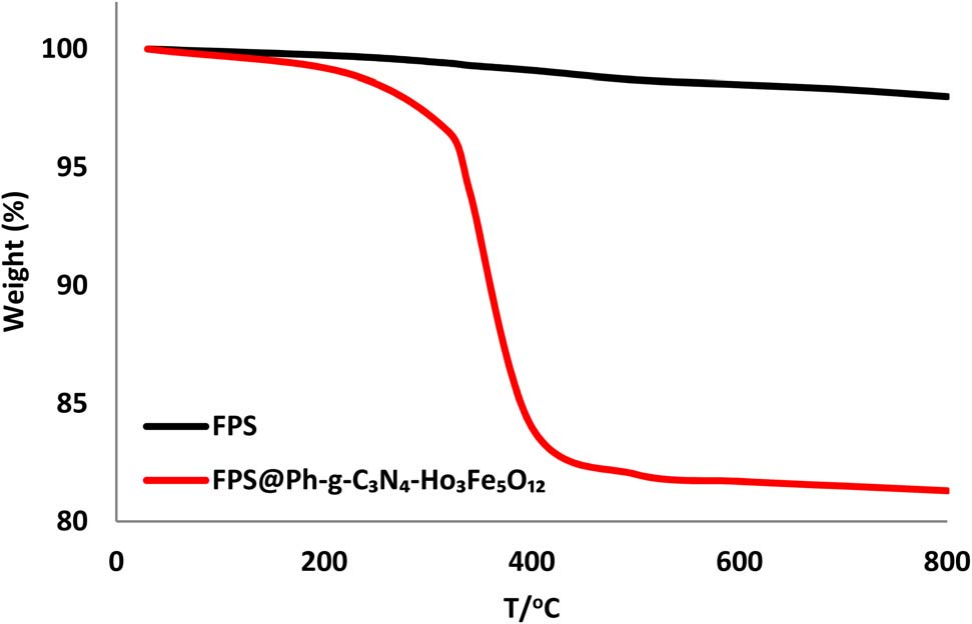
Thermogravimetric Analysis (TGA) profiles of FPS and FPS@Ph-g-C_3_N_4_-Ho_3_Fe_5_O_12_ nanocatalysts.

Thermogravimetric analysis (TGA) of the as-prepared, non calcined FPS@Ph-g-C_3_N_4_-Ho_3_Fe_5_O_12_ sample revealed a distinct two-step mass loss pattern. The first stage, occurring between 30 and 290 °C, exhibited a minor weight reduction of approximately 2–4%, which is attributed to the desorption of physisorbed and weakly bound water molecules from the catalyst surface. A second and more significant weight loss, approximately 16–18%, was observed in the temperature range of 260–380 °C, which can be attributed to the thermal decomposition of the Ph-g-C_3_N_4_ phase along with concurrent structural evolution toward the formation of the FPS@Ph-g-C_3_N_4_-Ho_3_Fe_5_O_12_ hybrid framework. This two stage mass loss profile indicates an initial release of volatile species followed by the progressive stabilization of the catalyst's crystalline structure (Fig. 5).

**Fig. 6:**
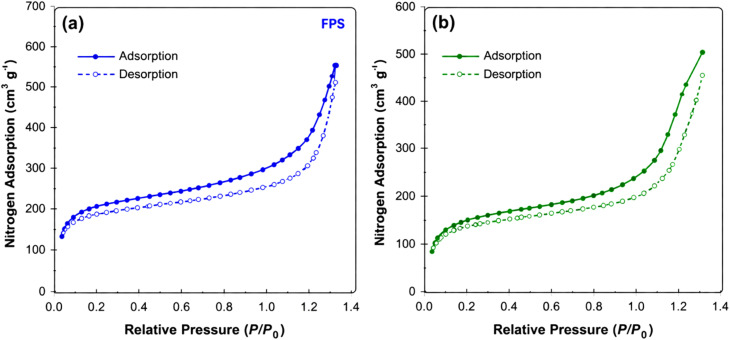
Adsorption–desorption isotherms of the (a) FPS; (b) FPS@Ph-g-C_3_N_4_-Ho_3_Fe_5_O_12_ MNPs.

Nitrogen adsorption–desorption measurements were carried out to evaluate the textural properties of the prepared materials. The Brunauer–Emmett–Teller (BET) surface areas were determined to be 612 m^2^ g^−1^ for pristine FPS and 502 m^2^ g^−1^ for FPS@Ph-g-C_3_N_4_-Ho_3_Fe_5_O_12_. The observed reduction in surface area after incorporation of Ho_3_Fe_5_O_12_ can be ascribed to partial pore occupation and blockage by the introduced ferrite nanoparticles. As shown in Fig. 6, the nitrogen adsorption–desorption isotherms of the FPS based materials display a typical type IV profile accompanied by a well defined H1 hysteresis loop, confirming the mesoporous nature of the structure. The pore size distribution, calculated from the desorption branch using the Barrett–Joyner–Halenda (BJH) method, exhibits a relatively narrow distribution centered around ∼13 nm (Table 1). Such uniform mesoporosity provides sufficient space for the accommodation and dispersion of Ho_3_Fe_5_O_12_ nanostructures within the FPS framework.

**Table 1:** Architectural attributes of FPS, and FPS@Ph-g-C_3_N_4_-Ho_3_Fe_5_O_12_ MNPs

Accelerators	*S*_BET_ (m^2^ g^−1^)	*V*_a_ (cm^3^ g^−1^)	*D*_BJH_ (nm)
FPS	612	3.1	13
FPS@Ph-g-C_3_N_4_-Ho_3_Fe_5_O_12_	502	2.4	7

**Fig. 7:**
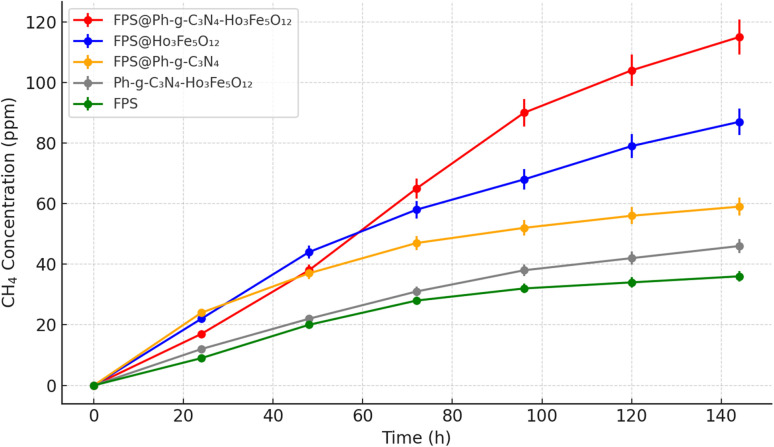
Comparison of CH_4_ production over time for various catalysts (FPS, Ph-g-C_3_N_4_-Ho_3_Fe_5_O_12_, FPS@Ph-g-C_3_N_4_, FPS@Ho_3_Fe_5_O_12_, and FPS@Ph-g-C_3_N_4_-Ho_3_Fe_5_O_12_) under visible light irradiation.

Fig. 7 illustrates the CH_4_ generation performance of FPS@Ph-g-C_3_N_4_-Ho_3_Fe_5_O_12_, FPS@Ho_3_Fe_5_O_12_, FPS@Ph-g-C_3_N_4_, Ph-g-C_3_N_4_-Ho_3_Fe_5_O_12_, and FPS under continuous visible-light irradiation for 144 hours. Measurements were taken every 24 hours. Among the tested samples, FPS@Ph-g-C_3_N_4_-Ho_3_Fe_5_O_12_ exhibited the highest CH_4_ production, while the other catalysts showed moderate to lower activity, with final CH_4_ concentrations of 114.39, 86.34, 58.06, 45.95, and 35.36 ppm, respectively.

The final detected CH_4_ concentrations for FPS, Ph-g-C_3_N_4_-Ho_3_Fe_5_O_12_, FPS@Ph-g-C_3_N_4_, FPS@Ho_3_Fe_5_O_12_, and FPS@Ph-g-C_3_N_4_-Ho_3_Fe_5_O_12_ were approximately 34.2, 43.9, 60.1, 82.7, and 119.2 ppm, respectively, after 144 hours of visible light irradiation. The CH_4_ production rate was determined using the equation:
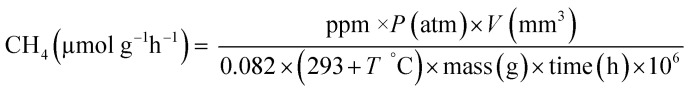


Among the tested photocatalysts, FPS@Ph-g-C_3_N_4_-Ho_3_Fe_5_O_12_ exhibited the highest CO_2_ conversion efficiency, significantly outperforming the individual and binary components. Its performance was approximately 3.5 times greater than FPS and nearly 2.8 times that of FPS@Ph-g-C_3_N_4_ alone. The conversion of CO_2_ into hydrocarbons, including C_1_ (*e.g.*, CH_4_) and C_2_^+^ species (*e.g.*, C_2_H_4_, C_2_H_2_, and C_2_H_6_), occurred with high selectivity, yielding approximately 42% CH_4_ and 58% C_2_^+^ products. The time resolved hydrocarbon distribution profiles provide compelling evidence supporting the multistep reaction mechanisms underlying the CO_2_ photoreduction process.

**Fig. 8:**
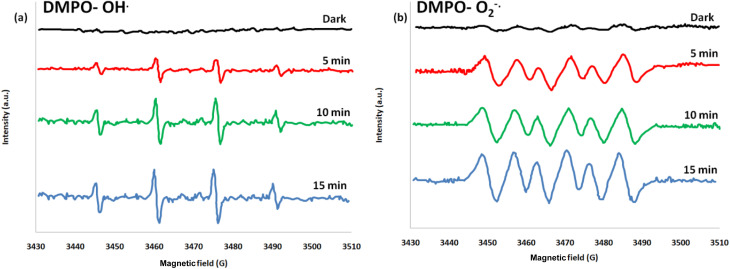
EPR spectra of DMPO spin-trapped radicals under visible-light irradiation: (a) DMPO–˙OH and (b) DMPO–˙O_2_^−^ obtained using FPS@Ph-g-C_3_N_4_-Ho_3_Fe_5_O_12_.

**Fig. 9:**
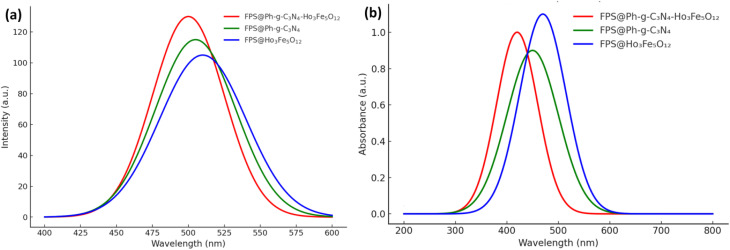
(a) Photoluminescence (PL) spectra, and (b) UV-vis absorption spectra of the catalyst and its individual components.

Fig. 8 displays the electron paramagnetic resonance (EPR) spectra of the FPS@Ph-g-C_3_N_4_-Ho_3_Fe_5_O_12_ suspension containing the DMPO spin trapping agent. The detection of DMPO–˙O_2_^−^ and DMPO–˙OH adduct signals in the presence of aqueous Na_2_CO_3_ confirms that ˙O_2_^−^ and ˙OH radicals act as crucial intermediates in the photocatalytic CH_4_ formation process. Under visible light irradiation, the EPR spectra revealed four (1 : 2 : 2 : 1) quartet patterns and six characteristic peaks corresponding to DMPO–˙OH (Fig. 9a) and DMPO–˙O_2_^−^ (Fig. 9a). The calculated hyperfine splitting constants for these adducts were *a*_N_ = 1.527 mT and *a*_Hβ_ = 1.462 mT for DMPO–˙OH, and *a*_N_ = 1.416 mT and *a*_Hβ_ = 1.251 mT for DMPO–˙O_2_^−^. These results demonstrate the significant role of reactive oxygen species in the photocatalytic CO_2_ to CH_4_ conversion over the FPS@Ph-g-C_3_N_4_-Ho_3_Fe_5_O_12_ hybrid catalyst.

**Fig. 10:**
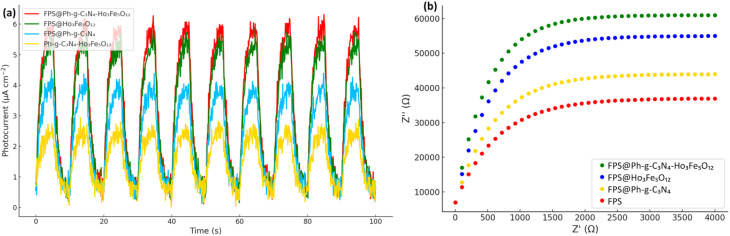
(a) Transient photocurrent response and (b) electrochemical impedance spectra (EIS) of the catalyst and its constituents, along with electron paramagnetic resonance (EPR) spectra.

The photochemical properties of the photocatalyst were systematically examined, with the results summarized here. The study investigated the photoluminescence behavior of FPS@Ph-g-C_3_N_4_-Ho_3_Fe_5_O_12_ within the wavelength range of 400–600 nm. Furthermore, the UV–visible absorption spectra of the catalyst components were analyzed, revealing that the observed absorption is primarily associated with the allowed optical transition between the electronic states in surface defect sites and the conduction band relative to the valence band. Additionally, particle sizes were estimated using the Brus equation and the effective mass approximation, based on their experimentally determined band gap values. These findings provide insight into the relationship between the optical response and the structural electronic characteristics of the photocatalyst (Fig. 9). The effective masses of electrons and holes in FPS@Ph-g-C_3_N_4_-Ho_3_Fe_5_O_12_, estimated at 0.21 *m*_e_ and 0.68 *m*_e_ respectively, were incorporated into the calculations along with the measured band gaps (*E*_g_ and *E*_np_), dielectric constant (*ε*), and particle radius (*R*) of the hybrid material. Photocurrent measurements, performed under intermittent visible light illumination, demonstrated stable and reproducible current responses for all tested photocatalysts, confirming efficient charge carrier generation and separation (Fig. 10). The integration of conductive FPS@Ph-g-C_3_N_4_-Ho_3_Fe_5_O_12_ into the composite architecture significantly prolonged electron lifetime and improved charge separation efficiency. Electrochemical impedance spectroscopy (*E*_IS_), widely employed to study semiconductor electrolyte interfacial properties, revealed that the hybrid catalyst exhibited the smallest Nyquist arc radius among all samples, indicating the fastest interfacial charge transfer. These results emphasize that high photocatalytic activity is governed not only by light absorption capability but also by effective charge separation and transport. The optical band gap (*E*_g_) values were calculated using the following equation:*αhν* = *A*(*hν* − *E*_g_)^*n*/2^

The absorption coefficient (*α*) and photon energy (*hν*) are key parameters in determining the energy bandgap of a material. By extrapolating the plot of (*αhν*)^1/2^*versus* hν, the bandgap energy (*E*_g_) can be estimated. Subsequently, the conduction band energy (*E*_cb_) can be calculated using the following relationship:*E*_cb_ = *E*_vb_ − *E*_g_

**Fig. 11:**
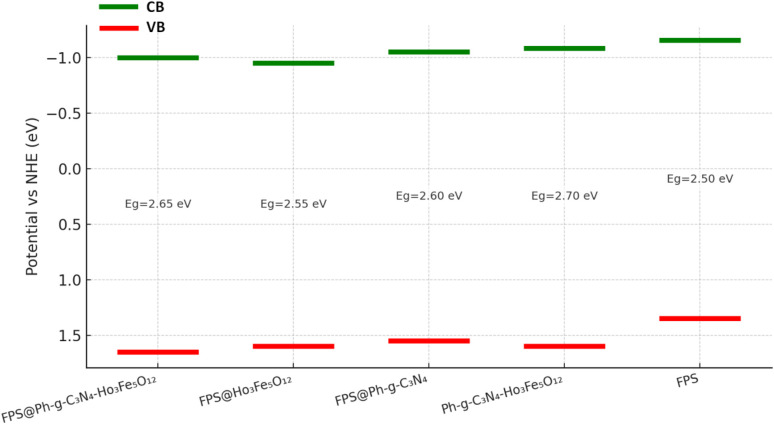
Band structure diagram of the catalyst and its constituents, along with electron paramagnetic resonance (EPR) spectra.

In Fig. 11, it is evident that the incorporation of FPS@Ph-g-C_3_N_4_-Ho_3_Fe_5_O_12_ nanocomposite markedly improved the photocatalytic performance. The conduction band edge (*E*_cb_) alignment indicates an enhanced capability for photoreduction, confirming that the engineered heterojunction facilitates efficient charge separation and transfer, thereby promoting superior CO_2_ conversion activity. The kinetic behavior of FPS@Ph-g-C_3_N_4_-Ho_3_Fe_5_O_12_ was evaluated using both pseudo first order and pseudo second order kinetic models. The pseudo first order model was applied assuming that the concentration of CO_2_ remains effectively constant under continuous flow conditions, and the reaction rate is mainly controlled by the photocatalytic surface processes. The pseudo first order model, describing the decrease in reactant concentration over time, is expressed as:1
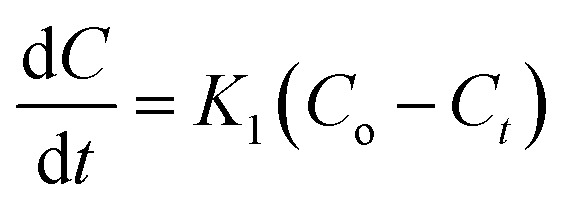
2
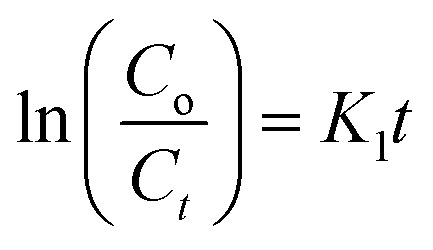


If a plot of ln(*C*_0_/*C*_*t*_) *versus t* yields a straight line with a negative slope, the slope represents the first order rate constant *k*_1_. The pseudo second order model is defined as:3
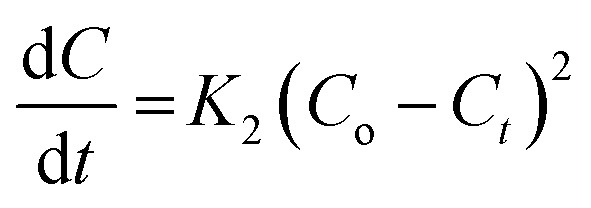
4
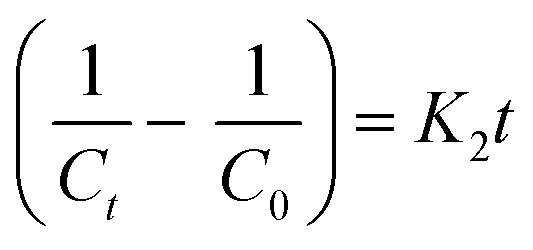


**Fig. 12:**
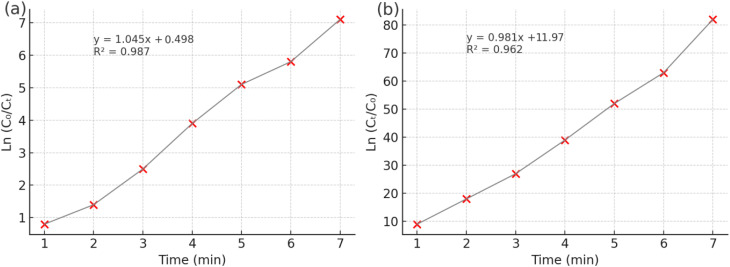
(a) First order kinetic analysis and (b) second order kinetic analysis of FPS@Ph-g-C_3_N_4_-Ho_3_Fe_5_O_12_ for CO_2_ photocatalytic reduction.

**Fig. 13:**
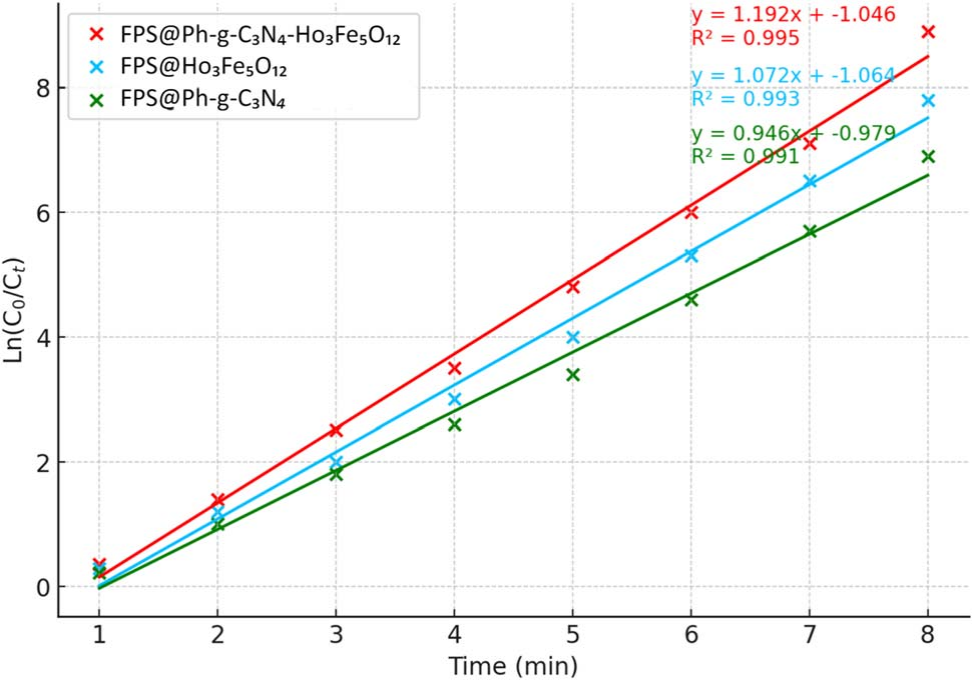
Kinetic analysis of pseudo first order reaction for CO_2_ photocatalytic reduction over FPS@Ph-g-C_3_N_4_-Ho_3_Fe_5_O_12_ under visible light irradiation.

**Fig. 14:**
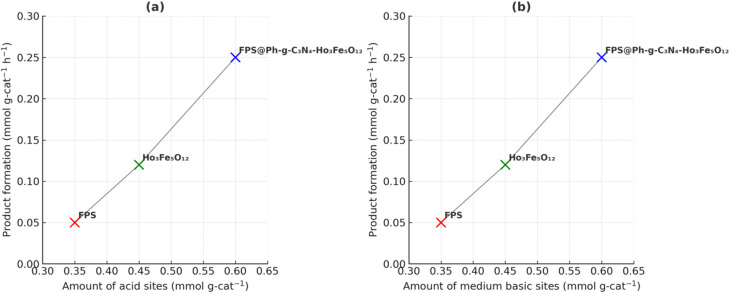
Correlation between product formation rate and (a) amount of acid sites and (b) amount of medium basic sites for FPS@Ph-g-C_3_N_4_-Ho_3_Fe_5_O_12_, Ho_3_Fe_5_O_12_, and FPS during CO_2_ photocatalytic reduction.

Experimental data indicated that *k*_1_ = 1.045 min^−1^ and *k*_2_ = 0.981 min^−1^, with the correlation coefficient (*R*_2_) of the pseudo first order model exceeding that of the pseudo second order model. The higher correlation coefficient (*R*^2^) obtained for the pseudo first order model compared to the pseudo second order model suggests that the reaction follows first order kinetics, where the rate is primarily dependent on the availability of active sites and reactant concentration. These results suggest that CO_2_ reduction over FPS@Ph-g-C_3_N_4_-Ho_3_Fe_5_O_12_ follows first order kinetics, with high selectivity toward hydrocarbon formation under visible light irradiation (Fig. 12). A straight line with slope *k* was obtained by plotting Ln(*C*_0_/*C*_*t*_) *versus* time for various catalysts in the CO_2_ photocatalytic reduction process. The analysis confirmed a pseudo first order reaction behavior (Fig. 13). The filamentous structure of the nanocatalyst played a key role, as it contributed to an increase in the rate constant. Among the tested catalysts, FPS@Ph-g-C_3_N_4_-Ho_3_Fe_5_O_12_ exhibited the highest rate constant, outperforming FPS, FPS@Ph-g-C_3_N_4_, FPS@Ho_3_Fe_5_O_12_, and Ph-g-C_3_N_4_-Ho_3_Fe_5_O_12_. Structural modification of FPS allowed for the formation of an interconnected network, enabling the stabilization of a larger amount of Ph-g-C_3_N_4_-Ho_3_Fe_5_O_12_ on the FPS surface. This enhancement increased the rate constant of CO_2_ photoreduction. The obtained rate constants were consistent with the results from inductively coupled plasma (ICP) analysis and hydrocarbon yield measurements. The relationship between active sites and the average reaction rate did not show a fully linear correlation, indicating that factors beyond acid–base properties also influence catalytic activity (Fig. 14).

**Fig. 15:**
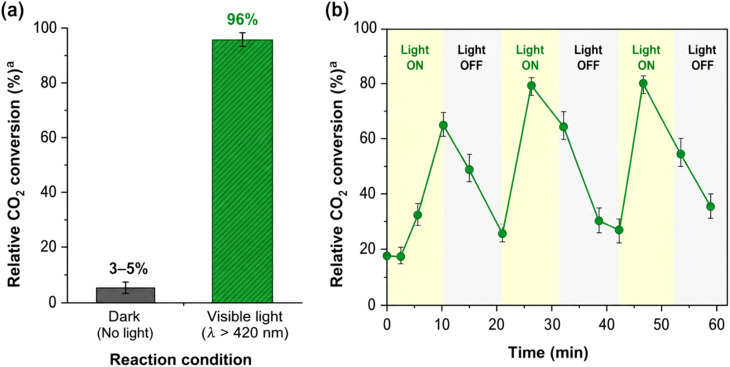
(a) Photocatalytic activity under dark and visible light (*λ* > 420 nm); (b) light on/off cycling test.

To rigorously verify the photocatalytic nature of CO_2_ reduction over the FPS@Ph-g-C_3_N_4_-Ho_3_Fe_5_O_12_ nanocomposite, control experiments were conducted under both illuminated and dark conditions. Under identical reaction conditions and continuous CO_2_ flow, only a negligible conversion of approximately 3–5% was observed in the absence of light, whereas a significantly higher yield of hydrocarbon products was obtained under visible light irradiation. This pronounced difference clearly indicates that the reaction is primarily driven by photoinduced processes rather than thermal effects. Furthermore, light on/off cycling experiments (Fig. 15) were carried out to monitor the dynamic response of the catalyst. The results demonstrate that hydrocarbon formation proceeds exclusively during illumination periods and is effectively suppressed in the dark, confirming that the catalytic activity is directly governed by photogenerated charge carriers. The efficient separation and transfer of these charge carriers within the FPS@Ph-g-C_3_N_4_-Ho_3_Fe_5_O_12_ heterostructure play a crucial role in activating CO_2_ molecules and promoting subsequent reduction pathways. Overall, these findings provide strong evidence that the observed CO_2_ conversion originates from a visible light driven photocatalytic process over the designed hybrid nanocatalyst.

A comparison with previously reported photocatalysts (Table 2) shows that the FPS@Ph-g-C_3_N_4_-Ho_3_Fe_5_O_12_ system exhibits improved performance under visible-light irradiation. This enhancement is attributed to the synergistic combination of the fibrous FPS support, conductive Ph-g-C_3_N_4_, and Ho_3_Fe_5_O_12_ ferrite, which together promote efficient charge separation, enhanced light absorption, and increased availability of active sites for CO_2_ activation. Although direct comparison is limited due to differences in experimental conditions and reporting units, the present results highlight the promising potential of this hybrid catalyst for photocatalytic CO_2_ reduction.

**Table 2:** Comparison of photocatalytic CO_2_ reduction performance of FPS@Ph-g-C_3_N_4_-Ho_3_Fe_5_O_12_ with previously reported catalysts under different irradiation conditions

Entry	Catalyst	Conditions (lamp, cutoff filter)	Product yield (µmol g^−1^ h^−1^)	Ref.
1	Ag-NPs/TiO_2_	200 W Hg	9.73	41
2	CuCo_2_S_4_@3B–TiO_2_	300 W Xe	42.2	42
3	Fe@TiO_2_/BCN	300 W Xe	24.7	43
4	Pd–Au/TiO_2_–WO_3_	300 W Xe	15.1	44
5	CdS/WO_3_	300 W Xe	3.75	45
6	R-ZnO@LDH	300 W Xe	11.4	46
7	FPS@Ph-g-C_3_N_4_-Ho_3_Fe_5_O_12_	Visible light (*λ* > 420 nm)	119.2 (ppm)	This work

**Fig. 16:**
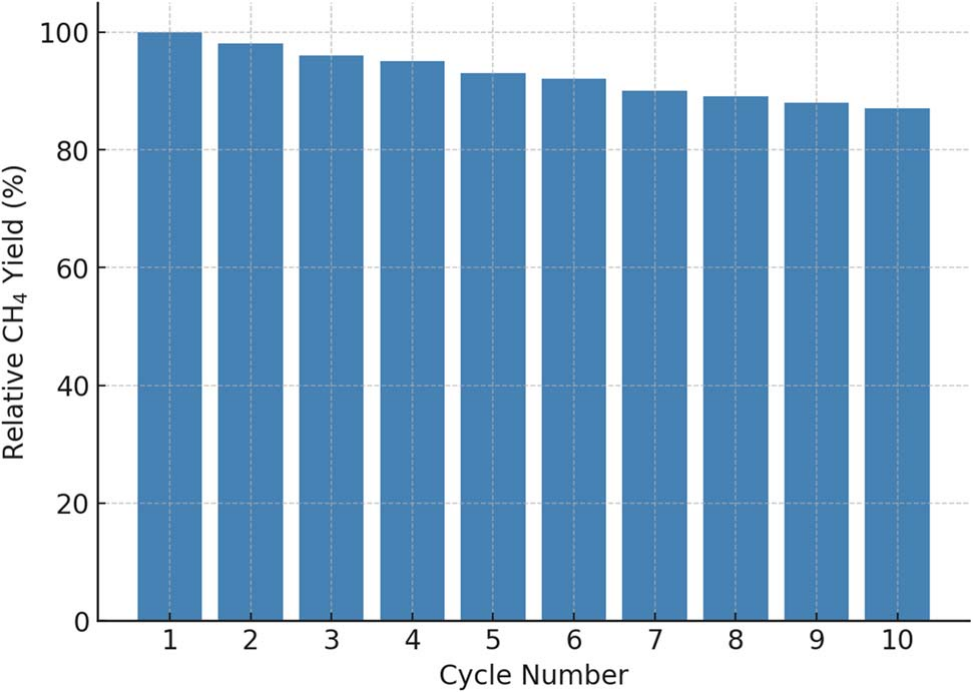
Recycling performance of FPS@Ph-g-C_3_N_4_-Ho_3_Fe_5_O_12_ over 10 consecutive CO_2_ photoreduction cycles under visible light irradiation, showing CH_4_ yield stability.

**Fig. 17:**
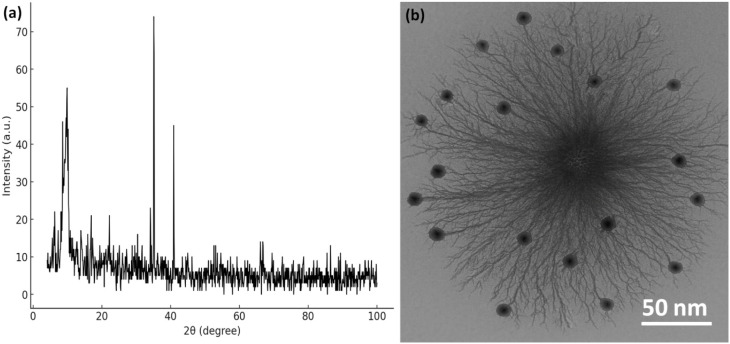
XRD pattern (a) and TEM (b) of recycled FPS@Ph-g-C_3_N_4_-Ho_3_Fe_5_O_12_ after ten photocatalytic cycles.

To evaluate the durability of FPS@Ph-g-C_3_N_4_-Ho_3_Fe_5_O_12_, the photocatalyst was subjected to ten consecutive CO_2_ photoreduction cycles under identical conditions (Fig. 16). After each cycle, the catalyst was magnetically separated from the reaction mixture using an external magnet, eliminating the need for centrifugation. The recovered material was washed with deionized water and ethanol, then dried before reuse. Only a slight reduction in photocatalytic efficiency was observed over repeated cycles, with a total decrease of approximately 9% after the tenth run, indicating excellent reusability. Structural analyses of the recycled catalyst using X-ray diffraction (XRD) and transmission electron microscopy (TEM) revealed no noticeable changes in crystallinity or morphology compared to the fresh sample. These results confirm that FPS@Ph-g-C_3_N_4_-Ho_3_Fe_5_O_12_ possesses high photostability, and the minor activity loss is likely due to gradual surface poisoning rather than structural degradation (Fig. 17).

## Conclusions

In this study, the nanocatalyst FPS@Ph-g-C_3_N_4_-Ho_3_Fe_5_O_12_ was successfully synthesized and applied as an efficient photocatalyst for the CO_2_ photoreduction reaction. Structural characterization using XRD, TEM, and complementary analyses confirmed that the fibrous-networked FPS structure, decorated with uniformly dispersed Ph-g-C_3_N_4_-Ho_3_Fe_5_O_12_ nanoparticles, provided a large interfacial contact area and effective electron transport pathways. Electrochemical impedance spectroscopy (*E*_IS_) and photocatalytic measurements revealed that the hybrid catalyst exhibited the lowest charge transfer resistance and the highest efficiency in separating photogenerated electron hole pairs among all tested samples. Band gap and conduction band position (*E*_cb_) analyses further demonstrated that structural modification of FPS improved the photocatalytic performance and enhanced the CO_2_ reduction capability under visible light. Kinetic investigations indicated that the CO_2_ photoreduction over this catalyst followed a pseudo first order model, with a significantly higher rate constant than those of its individual and binary counterparts. The catalyst maintained its activity over ten consecutive cycles, with only a slight reduction in performance. Its magnetic nature enabled easy separation from the reaction medium using a magnet, while post reaction XRD and TEM analyses confirmed the preservation of its structural integrity. Overall, these results demonstrate that FPS@Ph-g-C_3_N_4_-Ho_3_Fe_5_O_12_ possesses high stability, reusability, and outstanding photocatalytic efficiency, making it a promising candidate for sustainable and green CO_2_ conversion processes aimed at producing valuable hydrocarbon fuels.

## Conflicts of interest

There are no conflicts to declare.

## Data Availability

The data supporting the findings of this study are available within the article.
